# Dosing Methods to Enable Cell-Based In Vitro Testing of Complex Substances: A Case Study with a PAH Mixture

**DOI:** 10.3390/toxics11010019

**Published:** 2022-12-26

**Authors:** Alexandra C. Cordova, Lucie C. Ford, Alan Valdiviezo, Alina T. Roman-Hubers, Thomas J. McDonald, Weihsueh A. Chiu, Ivan Rusyn

**Affiliations:** 1 Interdisciplinary Faculty of Toxicology, College of Veterinary Medicine and Biomedical Sciences, Texas A&M University, College Station, TX 77843, USA; 2 Department of Veterinary Physiology and Pharmacology, College of Veterinary Medicine and Biomedical Sciences, Texas A&M University, College Station, TX 77843, USA; 3 Departments of Environmental and Occupational Health, Texas A&M University, College Station, TX 77843, USA

**Keywords:** passive dosing, human, bioactivity, high-throughput, extraction

## Abstract

Cell-based testing of multi-constituent substances and mixtures for their potential adverse health effects is difficult due to their complex composition and physical–chemical characteristics. Various extraction methods are typically used to enable studies in vitro; however, a limited number of solvents are biocompatible with in vitro studies and the extracts may not fully represent the original test article’s composition. While the methods for dosing with “difficult-to-test” substances in aquatic toxicity studies are well defined and widely used, they are largely unsuited for small-volume (100 microliters or less) in vitro studies with mammalian cells. Therefore, we aimed to evaluate suitability of various scaled-down dosing methods for high-throughput in vitro testing by using a mixture of polycyclic aromatic hydrocarbons (PAH). Specifically, we compared passive dosing via silicone micro-O-rings, cell culture media-accommodated fraction, and traditional solvent (dimethyl sulfoxide) extraction procedures. Gas chromatography-tandem mass spectrometry (GC-MS/MS) was used to evaluate kinetics of PAH absorption to micro-O-rings, as well as recovery of PAH and the extent of protein binding in cell culture media with and without cells for each dosing method. Bioavailability of the mixture from different dosing methods was also evaluated by characterizing in vitro cytotoxicity of the PAH mixture using EA.hy926 and HepG2 human cell lines. Of the tested dosing methods, media accommodated fraction (MAF) was determined to be the most appropriate method for cell-based studies of PAH-containing complex substances and mixtures. This conclusion is based on the observation that the highest fraction of the starting materials can be delivered using media accommodated fraction approach into cell culture media and thus enable concentration-response in vitro testing.

## 1. Introduction

Exposures to complex substances and mixtures are of concern not only to the environment but also to human health, especially during and after natural disasters where redistribution of contaminants is likely [1]. Conventional risk assessment paradigms typically focus on individual chemicals; evaluation of mixtures and complex substances remains a major challenge for regulators [2,3,4]. Accumulating evidence shows that individual-chemical assessments may underestimate chemical risks, even when dose reconstruction models attempt to account for chemical interactions in a mixture [5,6]. Recent improvements in mixtures risk assessment include nontargeted analytical methods and in vitro testing. On the one hand, advanced analytical chemistry methods are now being used to improve characterization of the chemicals in mixtures [7,8]. On the other hand, a number of new in vitro methods have been proposed to evaluate potential human health hazards of mixtures and complex substances [7,9]. Cell-based studies allow for high-throughput testing of both defined [10] and environmental [11] mixtures. Analyses of dose reconstruction of mixture effects based on in vitro data have concluded that model-based predictions of mixture effects from the individual components are often under-predictive [10,12]. 

Testing of whole mixtures, rather than their select components, is an approach that is gaining momentum in experimental toxicology [13]. However, delivery of complex substances for in vitro testing faces several major challenges: poor solubility, representativeness of the extract as compared to the entire substance including its hydrophobic constituents, compatibility with small testing volumes, stable exposure for the duration of the assay, and the presence of cell culture media components that may differentially bind individual mixture components. Most in vitro studies are based on dimethyl sulfoxide (DMSO) extraction following ASTM IP346 method [14] as a delivery vehicle for chemicals into cell culture media because it dissolves both polar and nonpolar compounds and is miscible with water. DMSO is also used for extraction of complex substances and environmental samples before bioactivity testing [14,15]. However, DMSO-soluble fractions of complex substances and mixtures may not represent the entire bioactive fraction of concern [16,17]. This limitation was recently highlighted in a decision from the European Chemicals Agency (ECHA) on a submission of in vitro bioactivity data [18] for petroleum substances extracted with DMSO in support of a grouping decision, stating that *“testing DMSO extracts does not provide a basis for reliably predicting the properties of the substance”* [19].

There are a number of widely used dosing approaches for aquatic toxicity testing of complex substances and mixtures, including solvent spiking, passive dosing, and dissolved (i.e., “accommodated”) fraction [20]. Solvent spiking involves direct addition of test substance diluted with a solvent of choice to the aquatic testing environment. Passive dosing involves submersion of a biocompatible polymer (e.g., O-rings) in neat test substance for absorption with subsequent transfer of the polymer and “loaded” substance into the testing environment, where equilibrium is established via passive diffusion [21,22,23,24,25]. Accommodated fractions are typically prepared by spiking neat substance into the same aquatic medium (e.g., water accommodated fraction, WAF) as the testing environment which is then stirred to stimulate compound partitioning to the WAF [21,26]. Unfortunately, these delivery methods, as described in OECD Guidelines, are not applicable to mammalian cell-based experiments because they are designed for much larger volumes and different testing media [27] than those used in high-throughput multi-well plate in vitro assays in mammalian cells; they also lack the supplements in cell culture medium which are known to exhibit differential, non-specific binding [16].

Several recent studies have sought to improve the dosing approaches by increasing throughput, testing compatibility with hydrophobic or very complex substances, or by applying them to mammalian cell-based assays. The use of silicone donor varieties to passively dose hydrophobic substances is the most common approach [21,22,23,24,25,28,29,30,31,32]. Many of these studies used polycyclic aromatic hydrocarbons (PAH) as surrogate hydrophobic compounds for understanding the toxicity of complex petroleum substances [33]. PAH of concern span a wide range of hydrophobicity, allowing researchers to understand differential partitioning to assay compartments and exposure stability over time [34,35]. While studies of dosing mixtures of PAH using various polymers have made several important advances, most of them were still focused on aquatic models, although some publications have used mammalian cells in 24-well plates [24,36,37,38]. Fewer studies have incorporated various WAF preparation schemes in high-throughput aquatic [29], or mammalian cell-based assays [39]. WAF preparations advantageously account for the complex composition of substances and differential solubilities of components in water as a medium [17]; however, it is often impossible or impractical to anticipate the partitioning behavior of individual constituents of concern from the time of WAF preparation to exposure in cell culture media.

**Figure 1:**
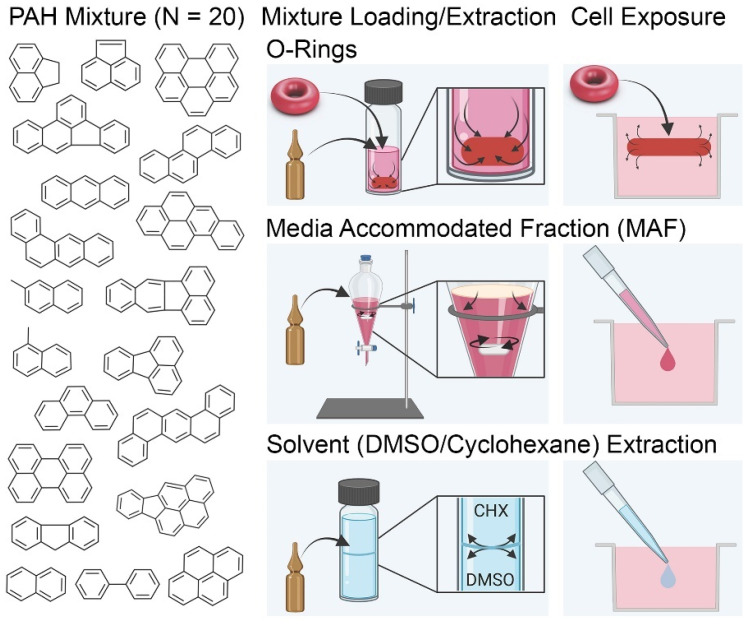
Overview of Experimental Design. Each dosing method was loaded with the standard mixture of PAH for 24 h. Prepared micro-O-Rings, MAF, and DMSO extract were then exposed to cell culture media with or without cells for up to 24 h. Analytical evaluation of PAH delivered to media and PAH free in media post-delivery were conducted to assess bioavailability to cells. Cytotoxicity was then conducted to measure potency of each method.

To further aid in the methodologies for testing complex substances and mixtures in mammalian cell-based systems, the present study sought to conduct a comparative analysis of dosing methods (Figure 1) to characterize the delivery of the individual components in a mixture setting in multi-well plate-based in vitro assays. Three methods were tested in parallel by adapting OECD Test Guidelines dosing methods to microscale volumes: passive dosing via silicone micro-O-rings, media-accommodated fraction, and solvent extraction with DMSO and cyclohexane. A reference mixture of 20 PAH was used as a representative complex substance for this study to represent a difficult-to-test hydrophobic mixture. We tested the delivery methods by varying concentrations tested and length of exposure and evaluated bioavailable and total concentrations in cell culture media using ultracentrifugation and quantitative GC-MS/MS analysis [16,40]. In addition, all dosing methods were applied to in vitro cell viability assays using two human cell lines.

## 2. Experimental Section

### 2.1. Chemicals and Biologicals

The analytical standard mixture of PAH was purchased from Absolute Standards Inc. (cat #60026, Hamden, CT, USA). PAH are a class of bioactive compounds of concern for environmental exposures and are major components of complex petroleum substances; model PAH such as benzo[a]pyrene are often used to characterize the risks of mixtures [41,42]. Of the twenty-five PAH in the commercial mixture, twenty were the substances of interest in this study based on the availability of the quantitative analytical methods (see below), these are listed in Table S1. Analytical internal standards (ISTDs) naphthalene-d8 (cat #48715-U, CAS # 1146-65-2), acenaphthene-d10 (cat #48417, CAS #15067-26-2) and perylene-d12 (cat #48081, CAS #1520-96-3) were purchased from Supelco (Supelco, Bellefonte, PA, USA) and included in all GC-MS/MS analyses. The internal standard assigned to each PAH of interest for relative quantification are listed in Table S2. Dulbecco’s Modified Eagle Medium (DMEM, cat #11965092, ThermoFisher, Waltham, MA, USA), Fetal Bovine Serum (FBS, cat #97068-085, VWR, Radnor, PA, USA) and penicillin/streptomycin mixture (cat #10378016, ThermoFisher, Waltham, MA, USA) were used to prepare MAF and in cell-based experiments. Silicone micro-O-rings (inner diameter 0.056”, outer diameter 0.176”, width 0.06”) were from McMaster Carr (cat #9396K61; Elmhurst, IL, USA). DMSO extracts were prepared with cell culture-grade DMSO (cat #sc-202581B, CAS #67-68-5, Santa Cruz Biotechnology, Santa Cruz, CA, USA) and cyclohexane (cat #C100307, Sigma-Aldrich, St. Louis, MO, USA). HPLC-grade methanol (MeOH, cat #34860), pentane (cat #34956), diethyl ether (Et2O, cat #309966), toluene (cat #650579), hexane (cat #650552) solvents were also purchased from Sigma-Aldrich, St. Louis, MO, USA. In vitro cytotoxicity assays were performed using CellTiter-Glo® 2.0 Luminescent Cell Viability Assay (cat #G9243, Promega, Madison, WI, USA) with EA.hy926 human endothelial cell line (cat #CRL-2922, ATCC, Manassas, VA, USA) and human HepG2 cell line (cat #HB-8065, ATCC, Manassas, VA, USA). Cells were cultured in 96-well black clear-bottom plates (cat #07-200-588; Corning, Kennebunk, ME, USA) and tetraoctylammonium bromide (TAB) was used as a positive control for the in vitro assays (cat #294136, CAS #14866-33-2, Sigma-Aldrich, St. Louis, MO, USA).

### 2.2. Analytical Instrumentation, Method, and Analysis

Targeted PAH analysis and quantitation was attained by 7890 GC coupled with 7010B GC/MS triple quadrupole mass spectrometer (Agilent Technologies, Santa Clara, CA, USA). All samples were prepared for analysis by adding 10 µL of a 10 µM solution of internal standards to 100 µL sample. Samples were then extracted using 50 µL methanol and 200 µL pentane:diethyl ether (1:1 *v*/*v*). Each sample was vortexed, mixed using a tissue homogenizer (Bead Ruptor 24, Omni International, Kennesaw, GA, USA) and centrifuged at 2600 rpm for 5 min [16,43]. The supernatant organic layer was pipetted into amber vials for GC-MS/MS analysis. The same protocol was applied for effluent samples from in vitro assays of 80 µL each, and were prepared using an equivalent ratio of internal standards (8 µL of 10 µM solution), methanol (40 µL), and pentane:diethyl ether (160 µL). The analytical method included Multiple Reaction Monitoring; 1 µL injection volume; pulsed splitless injection mode; DB-5ms Ultra Inert column, 60 m × 250 µm × 0.25 µm (122-5562UI, Agilent Technologies, Santa Clara, CA, USA); electron impact ionization source; 4 mL/min helium flow rate; 320 °C injection temperature; 320°C source temperature; 180 °C MS quad temperature; 300 °C transfer line temperature; and 18.5 psi nitrogen collision gas pressure. The oven gradient began at 80°C for 1 minute, increasing by 25 °C/min to 200 °C then increasing by 8 °C/min to 335 °C with 6.3 min hold for a total run time of 29 min, post-run time of 1.5 min, and equilibration time of 0.5 min [44]. Information on compound retention times and transitions can be found in Tables S2 and S3, respectively.

### 2.3. Preparing Micro-O-Rings

All micro-O-rings were cleaned before use by soaking in excess methanol (fully covered in a capped glass vial) for 2 h while shaking at 150 rpm, then transferred to a clean vial and soaked in fresh excess methanol overnight while shaking at 150 rpm. Micro-O-rings were then rinsed with Milli-Q water overnight, excess liquid was removed with a lint-free KimWipe, and samples dried for 24 h at room temperature [45]. Prior to use in cell culture experiments, micro-O-rings were sterilized by autoclaving for 30 min. Loading test solutions were prepared by spiking neat standard mix into methanol. Methanol is a solvent suitable for hydrophobic and hydrophilic compounds and was thoroughly washed from the micro-O-rings using Milli-Q water for 3 h in 1 h increments [24]. For all assays involving micro-O-ring exposures, the O-rings were submerged completely in test medium for up to 24 h.

### 2.4. Preparing Media Accommodated Fraction (MAF)

Fischer and coworkers recently demonstrated that increased serum content in cell culture media can compensate for losses in freely available concentration of reversibly bound hydrophobic chemicals, termed “serum-mediated passive dosing” [46]. We thus propose the use of cell culture media in place of water to prepare a media accommodated fraction (MAF). For analytical experiments, MAF were prepared at 10 μg/mL by adding 25 µL neat standard PAH mixture to 4.975 mL complete DMEM cell culture medium (media with 10% FBS and 1% penicillin/streptomycin solution) in a sterilized 10 mL glass separatory funnel. A sterilized magnetic stir bar was included in all MAF preparations and separatory funnels were stoppered. MAF was stirred for 24 h at room temperature by aligning the funnel with a stir plate [26]. MAF was then transferred directly out of the funnel for exposure into sterile vials. For exposures involving cells, MAF was prepared at 2× (20 µg/mL) the desired exposure concentration (10 µg/mL). The 2× MAF was then transferred to the working plate and diluted in log-scale from the highest concentration (20 µg/mL) for concentration response treatments.

### 2.5. Preparing DMSO Extracts

DMSO extracts were prepared following the standardized ASTM IP346 method [47]. Extracts were prepared by processing 100 µL neat PAH standard mix (2000 µg/mL) with 1 mL cyclohexane and 1 mL DMSO/cyclohexane (10:1) [16,18]. This step was repeated to yield 2 mL of DMSO extract of 75 µg/mL. Molar concentrations of each PAH in the standard mixture can be found in Table S1. Extracts were diluted in DMSO/cyclohexane (10:1) to ensure final concentration of 0.5% (*v*/*v*) DMSO/cyclohexane (10:1).

### 2.6. Testing Loading Kinetics of Micro-O-Rings

Loading efficiency of the micro-O-rings was evaluated following a modified protocol detailed by Smith and coworkers [24] (Figure S1). Briefly, three 4 mL replicates of 10 µg/mL solution of PAH standard mix were prepared in methanol. Seven clean micro-O-rings were added to each replicate vial for loading in excess solution up to various time points (0, 0.5, 1, 2, 4, 8, 24 h). “Excess” loading solution was defined by at least 10 mL:1 g ratio of loading solution:size of micro-O-ring [36]. At each time point, one micro-O-ring was removed from each vial and washed in a small volume of Milli-Q water for one hour. The washing was repeated three times for a total of 3 h. Individual micro-O-rings were then placed in autosampler vials with fused glass inserts containing 150 µL MeOH to simulate the working volume of a 96-well cell culture plate. Extraction was conducted for 24 h at ambient conditions and subsequently quantified by GC-MS/MS.

Data analysis of loading kinetics was conducted following the methods of Smith et al. [24]. Nominal concentration absorbed into the micro-O-rings C_silicone_ (µg/mL) was estimated by calculating a ratio of peak areas for each PAH detected (PAH Peak Area) at each time point (t) to the peak area for the same compound detected in the original loading solution (PAH Peak Area_0_) (Equation (1)). This ratio was then normalized to the initial concentration of the mixture.

(1) $$Nominal\;C_{silicone}=\frac{PAH\;Peak\;Area_{t}}{PAH\;Peak\;Area_{0}}*10\;µg/\mathrm{mL}$$

One-phase association in Prism software (GraphPad, San Diego, CA, USA) was used to determine first-order kinetics loading for each PAH (Equations (2) and (3)) [24].

(2) $$Y=Y_{0}+\left(Plateau-Y_{0}\right)*\left(1-exp^{-kx}\right)$$

(3) $$C_{silicone\left(t\right)}=C_{silicone\left(eq\right)}\left(1-exp^{-k_{loading}t}\right)$$

### 2.7. Assessing Recovery over Time from Exposure to Cell Culture Media without Cells

Recovery over time and optimal exposure time was evaluated using complete DMEM identical to that used for in vitro assays. After preparing each method (see above), exposure was conducted as follows. Eighteen micro-O-rings were loaded in 10 µg/mL PAH solution in methanol for 24 h, washed with Milli-Q water, and dried with a lint-free Kimwipe. MAF (10 µg/mL total) and DMSO (0.375 µg/mL total) treatments were also prepared as detailed above. Treatments were each exposed in a total volume of 2 mL DMEM in three separate replicate Eppendorf tubes to mimic plastic 96-well plates. Micro-O-ring treatments included 6 micro-O-rings per 2 mL (following Smith et al. 2010) [24], while MAF treatments were prepared as a dilution scheme (0%, 25%, 50%, 75%, and 100% MAF); DMSO extract was exposed at 0.375 µg/mL total. Treatments were incubated at 37°C while shaking at 550 rpm. Aliquots of 100 µL were taken for analysis from each replicate tube at prescribed timepoints: 0, 8, and 24 h for micro-O-rings; and 0, 4, 8, and 24 h for MAF and DMSO extract. Individual replicate aliquots were then transferred to 1.5 mL microcentrifuge tubes for extraction and GC-MS/MS analysis.

Nominal recovery (ng/mL) was calculated by normalizing the ISTD response ratio at respective time points t to the initial nominal concentration of individual PAH at time zero [PAH]_0_ (Equation (4)). For example, solvent extract was exposed at a mixture concentration of 0.375 µg/mL, or 15 ng/mL of individual PAH.

(4) $$Nominal\;Recovery_{t}=\frac{ISTD\;Resp.Ratio_{t}}{ISTD\;Resp.Ratio_{0}}*{\left[PAH\right]}_{0}\;$$

### 2.8. Assessing Protein Binding Behavior from Exposure to Cell Culture Media without Cells

Protein binding behavior of the PAH mixture was evaluated by simulating exposure conditions to be used in in vitro assays. Micro-O-rings were prepared as previously described, but to more closely mimic micro-O-ring exposure in 100 µL media per well for in vitro assays, 13 micro-O-rings were exposed in 1.3 mL complete DMEM (1 micro-O-ring/100 µL of media). Treatments were incubated for 1 or 24 h at 37 °C. Three 100 µL aliquots were then transferred to 1.5 mL microcentrifuge tubes for extraction and GC-MS/MS analysis. Of the remaining exposure media, three 300 µL aliquots were transferred to individual polycarbonate ultracentrifuge tubes (cat #343776, Beckman Coulter, Indianapolis, IN) for isolation of the unbound fraction. Ultracentrifuge tubes were balanced to within 0.0010 g difference and spun using an Optima MAX-XP Ultracentrifuge (Item #393315; Beckman Coulter, Brea, CA, USA) at 90,000 rpm for 4.5 h at 4 °C. Once ultracentrifugation was complete, 100 µL each sample was aliquoted to clean microcentrifuge tubes and extracted for GC-MS/MS analysis (Figure S1). For MAF, samples were prepared following the same procedure. After loading, separate samples were aliquoted from each MAF for analysis of media-dissolved fractions and unbound fractions, respectively. For each, several dilutions were tested: 0%, 25%, 50%, 75%, and 100% MAF [33]. These were prepared by diluting MAF in appropriate amounts of complete DMEM for a total volume of 2 mL. Dilutions were then vortexed and incubated for 1 or 24 h at 37 °C. Three 100 µL aliquots were then transferred to individual tubes for extraction and analysis, while three 300 µL aliquots were transferred to ultracentrifuge tubes for unbound fraction (F_UB_) analysis.

For DMSO extract, samples were exposed at 0.375 µg/mL by aliquoting appropriate amounts of 75 µg/mL extract into 2 mL total volume DMEM. This concentration was chosen to simulate the highest possible concentration achievable in vitro while maintaining DMSO content at 0.5%. Samples were vortexed and incubated for 1 or 24 h at 37 °C. Aliquots were then transferred for analysis and ultracentrifugation, respectively.

### 2.9. Protein Binding Analysis of the Media-Dissolved Fraction and Unbound Fraction

Quantitation of the media-dissolved fraction (F_MD_) and F_UB_ of test substance was performed [16]. The internal standard response ratio of each PAH was compared to that from a stock solution prepared in pentane/diethyl ether (1:1 *v*/*v*) equivalent to the concentration of each treatment (Equation (5)). Stock was analyzed and run with each batch of samples to obtain response ratios representative for that batch (Figure S4).

(5) $$F_{MD}{\;\mathrm{or}\;F}_{UB}\;\left(\%\right)=\frac{Responseratio\left(PAH\;in\;sample\right)}{Responseratio\left(PAH\;in\;stock\right)\;}$$

### 2.10. In Vitro Experiments

Human endothelial (EA.hy926) and hepatoma (HepG2) cells were selected for compatibility with DMEM media and to test the bioavailability of PAH mixture in various dosing protocols. Cells were cultured in black flat bottom 96-well plates using a complete DMEM media (DMEM + 10% FBS + 1% penicillin/streptomycin). EA.hy926 cells were seeded at 33,000 cells per well in a volume of 50 µL [48]. HepG2 cells were seeded at 25,000 cells per well in a volume of 50 µL [49]. Overall, four different experiments were run: two experiments with EA.hy926 cells and two experiments with HepG2 cells. For each cell type, one experiment exposed cells to the three dosing methods for 1 h while the other exposed cells to identical conditions for 24 h. Each experiment was conducted on a single plate, with four replicates per concentration of each condition. At least four replicates were also included for positive and negative controls. PAH mixture delivery was conducted as follows. Micro-O-rings were loaded with test substance for 24 h and transferred directly to the cell culture assay plate using sterile tweezers. MAF was prepared as detailed above and then transferred to a working plate, where concentrations were diluted to achieve 2× concentrations desired in the assay plate. MAF was exposed at a highest concentration of 10 µg/mL (prepared as 20 µg/mL) of the mixture based on concentrations yielding best-quality chromatography in analytical assays (data not shown) and following solubility estimations in media [46]. Each concentration was diluted to achieve 10-fold dilutions of MAF in independent wells. DMSO extracts were prepared with a desired assay plate exposure of 0.5% DMSO. From initial preparation of the 75 µg/mL extract, individual solutions of 0.75, 7.5, 75, and 750 ng/mL were prepared for the 2× working plate, each at 1% DMSO in sterile DMEM. Final exposure concentrations in the assay plate were 0.375, 3.75, 37.5, and 375 ng/mL in 0.5% DMSO. Figures and schematics throughout this work reference the highest of these exposure concentrations as 80% of total because the GC-MS/MS method used was tailored for 20 out of the 25 equi-weight compounds in the PAH mixture. Total volume per well was maintained at 100 µL throughout all in vitro experiments.

Cytotoxicity was characterized using the CellTiter-Glo® 2.0 Luminescent Cell Viability Assay and luminescence was read using FLIPR Tetra with Screenworks 4.0 (Molecular Devices, San Jose, CA, USA). For each analysis, 80 µL treatment media was removed for analytical quantification and wells were replenished with 30 µL maintenance media and 50 µL CellTiter-Glo® reagent (added in equal volume to each well). The plates were mixed at 600 rpm for 2 min, then protected from light for 10 min before running the luminescence assay [50].

Cytotoxicity data were normalized to treatment-specific vehicle controls from each dosing method. DMSO treatments were normalized to the average response of control wells treated with DMEM and 0.5% DMSO/cyclohexane (10:1 *v*/*v*). MAF treatments were normalized to untreated wells containing only DMEM. O-Ring treatments were normalized to wells treated with O-Rings loaded with and washed of MeOH (0 µg/mL PAH mix). Normalized data was then fitted to [inhibitor] vs. normalized response curve in GraphPad (Prism Software, San Diego, CA, USA) software and scaled to % cell viability. Statistical analyses were performed in GraphPad and are detailed in the figure legends.

### 2.11. Quantification of Media-Dissolved Fraction from In Vitro Samples

After exposure but prior to assay, 80 µL treatment media was removed from each well in the assay plate and transferred to individual Eppendorf tubes for extraction. Each sample underwent liquid-liquid extraction and GC-MS/MS analysis (see above) for quantification of media-dissolved fraction. Unbound analyses were not performed due to small volumes of media per well.

## 3. Results and Discussion

### 3.1. Micro-O-Ring Loading of PAH

Previous studies applied passive dosing techniques in vitro to examine dosing efficiency compared to solvent spiking; however, these studies focused on exposure of a single chemical (benzo[a]pyrene) and were conducted using large volume (i.e., 24-well) cell culture plates [36,37]. The present study tested a mixture of 20 PAH of varying LogK_OW_ to represent a complex substance. Micro-O-rings loaded in a methanol solution of the PAH mix were evaluated at 0, 0.5, 1, 2, 4, 8, and 24 h to investigate loading efficiency of the small absorption reservoirs (Figure S1). Micro-O-rings were selected based on size compatibility with 96-well plates and had an outer diameter that was ~3× smaller than those used by Smith et al. [24], as their experiments were conducted in 24-well plates. In addition, a more recent study conducted by Heger et al. [29] used O-rings to passively dose complex PAH-containing substances to cells in 96-well plates with approximately the same outer diameter as those used herein; however, their study did not characterize the kinetics of absorption/release of the PAH.

**Figure 2:**
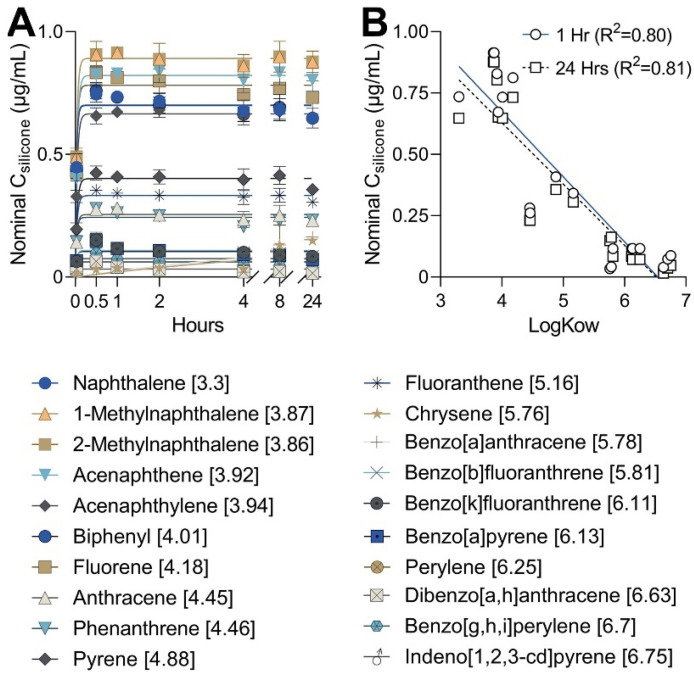
(**A**) GC-MS/MS nominal quantification of micro-O-ring absorption of each PAH in a 10 µg/mL methanol solution based on modified protocol from Smith et al. with corresponding LogK_OW_ values in brackets [24]. Absorption was measured at 0, 0.5, 1, 2, 4, 8, and 24 h timepoints. All PAH are absorbed to saturation quickly (within 1 hr) except for chrysene and benzo[a]anthracene, which are structural isomers. Other structural isomers also exhibit comparable kinetics of absorption (Table S4). (**B**) Correlation plot showing the negative inverse relationship between LogK_OW_ of individual PAH and the nominal concentration absorbed by micro-O-rings after 1 and 24 h. Results over 1 and 24 h exhibit similar relationships to hydrophobicity, indicating that saturation is reached quickly and maintained.

Micro-O-rings used herein demonstrated relatively rapid and consistent loading from methanol solutions, occurring within the first 30 min for most compounds (Figure 2A). The two exceptions to this trend were chrysene and benzo[a]anthracene, which are isomers and have very similar loading rates. All isomeric compounds were absorbed at nearly equal rates; for example, 1-methylnaphthalene and 2-methylnaphthalene both had best-fit rate constants of 26.7 h^−1^. Rate constants were higher in the current study than those reported for PAH by Smith et al. [24] because of the differences in O-ring size/volume. However, the ratio of PAH rate constants compared to that of naphthalene were similar for most compounds between Smith et al. [24] and our results (Table S4). More detail depicting the behavior of individual PAH from Figure 2 can be found in Supplemental Information, along with the rate constants calculated for each PAH (Figure S2, Table S4). In concordance with previous findings, a strong inverse linear correlation (R^2^ = 0.8) was observed between the hydrophobicity of the PAH (LogK_OW_) and the nominal concentration absorbed into the micro-O-rings [24]. The correlations were similar between 1 h (R^2^ = 0.8) and 24 h (R^2^ = 0.8) timepoints, indicating little change in absorption over time (Figure 2B).

### 3.2. Testing of Dosing Methods in Complete Media without Cells: Analysis of Recovery from Cell Culture Media over Time

**Figure 3:**
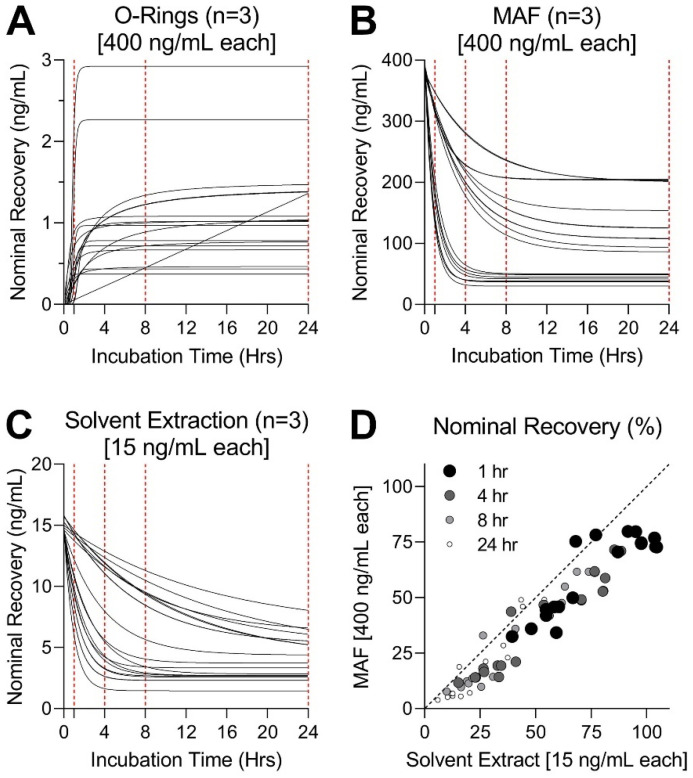
(**A**) Nominal recovery (ng/mL) from exposure of micro-O-Rings loaded with 400 ng/mL each PAH in mixture ([10 µg/mL] total) to media without cells measured at 1, 8, and 24 h (red dashed lines). Stability in recovery from micro-O-rings was observed for most compounds after 8 h. (**B**) Nominal recovery (ng/mL) from exposure of MAF prepared with 400 ng/mL each PAH, [10 µg/mL] total, to media without cells measured at 1, 4, 8, and 24 h. Longer exposure times yielded less recovery, stabilizing by 8 h for most compounds. (**C**) Nominal recovery (ng/mL) from exposure to DMSO extract prepared with 15 ng/mL each PAH, [0.375 µg/mL] total, to media without cells measured at 1, 4, 8, and 24 h. Recovery stabilized by 8 or 24 h for most compounds. (**D**) Strong positive linear correlation observed between fractional recovery from DMSO extract (x-axis) and MAF (y-axis) exposures to media over 1 (R^2^ = 0.81), 4 (R^2^ = 0.91), 8 (R^2^ = 0.93), and 24 h (R^2^ = 0.89) timepoints (varied by size and shade of gray).

Next, we tested recovery of PAH from three dosing methods by exposing test mixtures to complete cell culture media without cells. These experiments were designed to determine the optimal exposure time needed to reach equilibrium. Time-course experiments were used to collect measurements from nominal amounts of each PAH extracted from complete DMEM media (Figure 3). In each dosing method, the PAH mixture was prepared using the maximum concentration possible for in vitro conditions; these varied because for solvent extractions, considerable dilution of the starting test material is unavoidable. Extraction with DMSO and cyclohexane was conducted to simulate extractions often used for in vitro exposure of more complex substances [11,15,16]. Because DMSO can exert toxicity to cells at concentrations exceeding 0.5% [51], test substances need to be further diluted before in vitro exposures take place. Overall, the starting PAH mixture was diluted to 75 µg/mL during extraction and then to 0.375 µg/mL final highest test concentration in vitro to achieve DMSO < 0.5%. The highest concentrations for preparation of MAF were based on modeled solubility in cell culture medium (Figure S3) [46]. The highest concentration soluble in DMEM was predicted to be 20 µg/mL of the mixture; MAF was therefore prepared at 10 µg/mL to replicate what exposure conditions would be after dilution in the assay plate. Micro-O-rings were loaded in 10 µg/mL PAH solution to enable direct comparisons to the experiments with MAF.

Desorption of PAH from each loading method into complete cell culture medium reached steady-state by 8 h for most tested substances, with all but chrysene reaching steady-state by 24 h (Figure 3A). It is noteworthy that overall, the micro-O-ring dosing method demonstrated the lowest nominal recovery as compared to either MAF or DMSO extraction, even though the O-rings were loaded with as high of an amount of each PAH as MAF. Rate constants and plateau values for micro-O-ring desorption are detailed in Supplemental Information (Table S5). The amount of PAH delivered by micro-O-rings was only about 50% of that delivered by DMSO extraction despite concentrations in the latter being more than 25-times lower. This indicates that micro-scale O-rings absorb relatively small amounts of the starting material because of their small volume of the polymer. It is also possible that the washing of micro-O-rings, a necessary step to remove methanol solvent, may also contribute to the overall loss of the test material.

The downward trends in time-course delivery of PAH to media in experiments with MAF (Figure 3B) and DMSO extracts (Figure 3C) demonstrate equilibration of compound partitioning over time to various compartments in the exposure system. These processes are known to include reversible non-specific binding to media components, sorption to tubes, volatilization, or cell uptake [34,35,46,52]. Total recovery from MAF exposure was higher than that from DMSO extraction. The highest recovery was observed for indeno[1,2,3-cd]pyrene (7 ng/mL for DMSO and 201 ng/mL for MAF) and benzo[g,h,i]perylene (4 ng/mL for DMSO and 200 ng/mL for MAF), while biphenyl (1 ng/mL for DMSO and 30 ng/mL for MAF) and both methylnaphthalene isomers (2.3 ng/mL for DMSO and 37 ng/mL for MAF) yielded the lowest recovery via both dosing methods (Table S5). We observed nearly identical fractional recovery of tested PAH across all timepoints for DMSO extraction and MAF as they were highly correlated (average R^2^ = 0.89) (Figure 3D).

### 3.3. Testing of Dosing Methods in Complete Media without Cells: Protein Binding Analysis of Media-Dissolved and Unbound Fractions

To evaluate protein binding efficiency of compounds tested, media samples exposed to each of the three dosing methods either underwent ultracentrifugation to determine F_UB_ or were immediately extracted to quantify F_MD_. Ultracentrifugation was previously shown to be an effective technique for evaluating protein binding of lipophilic compounds in human plasma and cell culture media as compared to rapid equilibrium dialysis and was therefore the technique selected for this study [53]. Evaluation of F_UB_ using solid-phase microextraction (SPME) in these small-volumes (50-100 µL) was difficult as compound recovery was below the limit of quantification (LOQ), defined as a signal-to-noise (S/N) ratio less than 10 (data not shown). Quantification of F_MD_ and F_UB_ for each test PAH was achieved using targeted GC-MS/MS analyses. F_MD_ was used as a measure of total chemical extractable from media for each dosing method. The F_UB_ was then determined from sample ultracentrifugation and comparison to stock solutions of equivalent nominal concentration.

**Figure 4:**
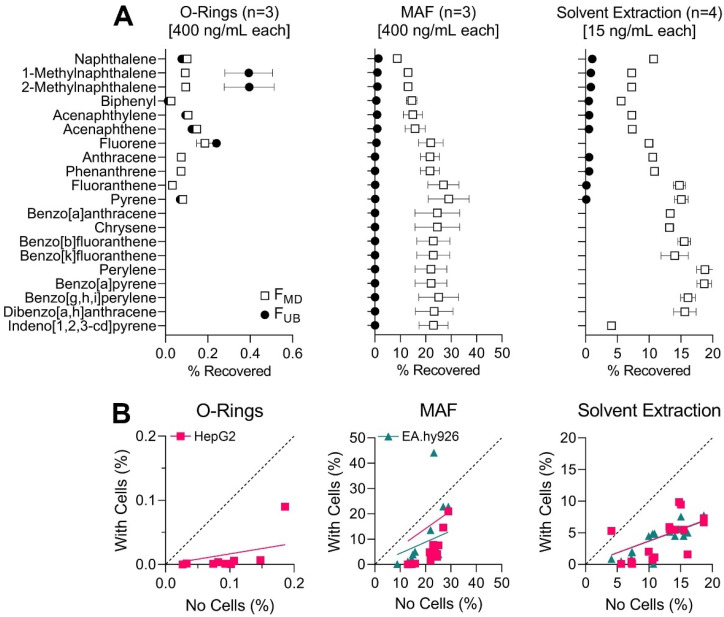
Compound-specific recovery from exposure of each dosing method to media without cells. Squares indicate media dissolved fraction (F_MD_) and circles indicate fraction unbound (F_UB_) for each PAH. Compounds are listed in order of increasing retention time (RT). PAH recovery below LOQ are shown as blank, while instances where internal standard was also below LOQ are not reported (NR). (**A**) Recovered F_MD_ and F_UB_ from exposure to micro-O-rings loaded with 0.375 µg/mL PAH solution for 1 hr, showing only smaller PAH are reliably recovered (left). Measurements after exposure for 24 h are included in Figure S4. Recovered F_MD_ and F_UB_ from exposure to 10 µg/mL MAF, showing the most consistent recovery across all compounds (center). MAF was prepared based on highest concentration possible within solubility of PAH in media calculated from Fischer et al. 2018 (Figure S3). Recovered F_MD_ and F_UB_ from media incubation with DMSO extract simulating highest possible in vitro exposure levels (right). Comparable F_MD_ between exposure to DMSO extract and MAF was observed for all compounds. (**B**) Correlation between exposures without (x-axis) and with (y-axis) cells show a higher overall recovery without cells for all dosing methods for HepG2 (pink squares) and EA.hy926 (green triangles) cell lines. Lowest percent of compounds recovered from exposure with cells relative to no-cell exposure was observed for O-ring dosing method (left). MAF yielded best correlation between exposures with and without cells (center). DMSO extract exposure with cells yielded most similar recovery between the two cell types, though less PAH was recovered than with MAF (bottom).

Figure 4A shows recovery of the individual PAH after 1 h exposure comparing F_MD_ and F_UB_ for each dosing method. In experiments with MAF, the highest average F_MD_ of all PAH (20.6%) was observed (Figure 4A center), closely followed by DMSO extracts (12.6%) (Figure 4A right), while micro-O-rings exhibited the lowest average uptake of all methods (0.093%) despite loading using the same mixture concentration as MAF (Figure 4A left). It should be noted that compounds quantified using perylene-d12 internal standard (benzo[a]anthracene and larger) were not quantifiable for F_UB_ arm of the DMSO extraction experiment and are not reported for the micro-O-rings experiment (both F_MD_ and F_UB_) because of the analytical issue with the standard and sample availability. Raw data for tested compounds and corresponding internal standards are provided in Table S6. After 24 h exposure, fewer compounds were detectable from micro-O-ring exposure, indicating lower bioavailability (Figure S4).

Overall, we found that MAF and DMSO extracts demonstrated greater uptake of PAH with three or more aromatic rings, which is consistent with previous observations [16]. Specifically, greater amounts of triaromatic (TriAr) and mid-size polyaromatic (PolyAr) PAH were measured from MAF and DMSO extract exposures as compared to diaromatic (DiAr) and naphthenic diaromatic (NDiAr) classes, regardless of the exposure time (Figure 4A and Figure S4). Because small PAH volatilize easily, lower recovery for DiAr and NDiAr was expected due to some volatilization during loading and exposure. Greater amounts of serum in media (10%) have been shown to counteract losses of hydrophobic compounds including PAH due to plate sorption partitioning; however, chemicals that exhibit high air-medium partitioning may not conform to these conclusions [54]. It is noteworthy that free (i.e., unbound) fraction was low for all delivery methods, consistent with previous reports that >90% of benzo[a]pyrene delivered into complete cell culture medium was bound to medium constituents [37]. Still, though comparable to F_MD_ for O-ring exposure, F_UB_ was approximately an order of magnitude lower than F_MD_ for exposures from MAF and solvent extract. Of the three delivery methods, MAF exhibited most consistent recovery for all PAH via both F_MD_ and F_UB_.

### 3.4. Application of Each Dosing Method to In Vitro Studies: Determination of Media-Dissolved Fraction after Incubation In Vitro

Because dosing methods tested herein are intended for in vitro experiments and cells in culture would serve as an additional compartment for delivered substances, we repeated experiments detailed above (Figure 4A) with cells present. Fractional recovery from each dosing method was determined and the data are reported in Figure S5. Figure 4B shows the correlations of the percent recovery values obtained from experiments with or without cells for each dosing method. As expected, all exposures to media alone yielded higher F_MD_ (indicated by the regression slopes being below the unit line in each panel of Figure 4B) than treatment effluents from cell culture exposures, representing the degree of chemical partitioning to the cells and how this differs for each cell type tested. Correlations between the ISTD response ratios of the two cell types can be found in Supplemental Information, along with F_MD_ of individual PAH compared between no-cell and in vitro assays for 1 and 24 h timepoints (Figure S5). Detailed assignment of internal standards to each PAH tested is also included in Supplemental Information (Table S2).

For micro-O-ring exposures, only the HepG2 cell-based experiment yielded acceptable chromatography for quantitative analysis. For HepG2 exposure, the correlation between cell and no-cell experiments (pink squares) demonstrates greater PAH recovery in no-cell assays, indicating 83% chemical partitioning to other compartments (Figure 4B left). Most comparable to this experimental set-up are studies of passive dosing via several types of polymer materials, including larger O-rings, discs, rods, or tubing of silicone or polydimethylsiloxane (PDMS) [21,24,25,30,36,37]. These experiments of passive dosing are most commonly applied to aquatic toxicity tests and usually are conducted on a large scale (e.g., vials or large bottles); fewer studies utilized 6- or 24-well plates, and only one study used 96-well plates [21,22,23,25,28,29,30,31,32,55,56,57,58,59]. Thus, our data show that while O-rings are a feasible donor on a large scale; this method is less preferred in micro-scale in vitro assays.

Recovery from MAF exposure yielded F_MD_ most closely correlated to F_MD_ extracted from no-cell experiments for both cell types (Figure 4B center). Based on the slope of best-fit regressions compared to unity, an estimated 56% of PAH exposed partitioned to other compartments, including cells, via testing with EA.hy926 cells and 29% partitioned elsewhere in testing with HepG2 cells. While MAF has not been previously applied specifically as a dosing method, serum-mediated passive dosing using DMEM has been tested as a preventative mechanism for plate sorption in various multi-well formats [46,54]. Fischer et al. [54] noted that over a span of 96 h, compounds with higher LogK_OW_ exhibited much faster depletion in the medium containing no protein supplement such that compounds preferentially bound to the plastic. However, when tested in cell-based assays including 10% FBS, they found 2.5% depletion of chemical in 96-well plates over 24 h [54].

Recovery from exposure to DMSO extract yielded the most similar F_MD_ between the two cell types compared to no-cell experiments (Figure 4B right). An estimated 63% of PAH exposed partitioned to other well compartments in testing with both EA.hy926 and HepG2 cells. This is comparable to results described by Chapman et al. after passive exposure of benzo[a]pyrene to MCL-5 cell type, showing only 0.033% present freely in cell culture medium, 93% bound to medium constituents, 4.2% bound to cells, and 2.6% bound to plastic [37].

### 3.5. Application of Each Dosing Method to In Vitro Studies: Cytotoxicity Assay

Finally, to determine whether observed differences in delivery of PAH through various dosing methods elicited differential effects on cells in vitro, we tested each dosing method with Ea.hy926 and HepG2 cell lines. Cell viability was used as a measure to determine which dosing method was most effective in delivering the PAH standard mixture in vitro. All methods were incubated with cells for 1 or 24 h. The highest nominal exposure concentration tested for the total mixture was 100 µg/mL for micro-O-rings, 10 µg/mL for MAF, and 0.375 µg/mL DMSO extract.

**Figure 5:**
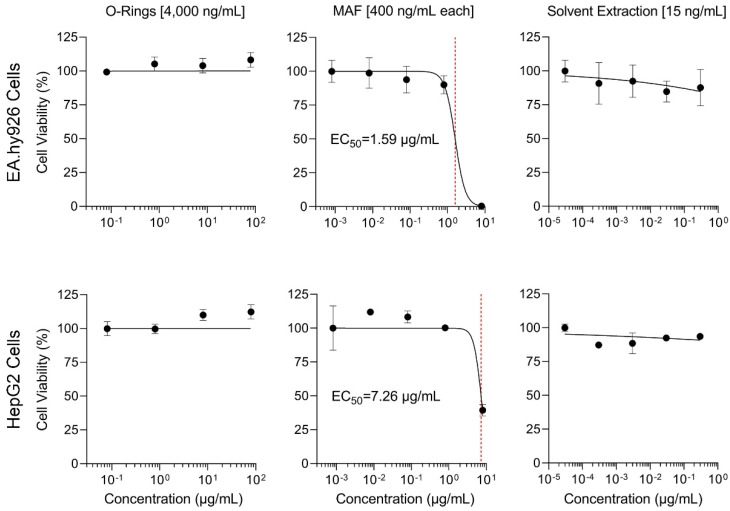
Concentration-response analysis of cytotoxicity of EA.hy 926 cells (**top**) and HepG2 cells (**bottom**) when exposed to each dosing method for 1 h with corresponding estimated EC_50_ values (red dashed lines): micro-O-rings (**left**), MAF (**center**), and DMSO extract (**right**). Concentration-response analysis for cytotoxicity assays conducted after 24 h incubation are included in Figure S6. All wells exposed to DMSO extracts, including negative controls, contained 0.5% DMSO/cyclohexane (10:1). Highest concentration tested using each method refers to the nominal concentration of each PAH of interest as exposed in the assay plate. Concentrations tested for micro-O-Rings refer to nominal concentrations of loading solutions prepared using 2000 µg/mL neat PAH mixture.

For both EA.hy926 and HepG2 cell lines, MAF was the only dosing method to elicit concentration-dependent cytotoxic effects (Figure 5). The concentration at which 50% of cells exhibited cytotoxicity (EC_50_) was used as a metric to compare potency between dosing methods. Exposures via DMSO extract and micro-O-rings exhibited little to no effect, even though micro-O-rings were loaded with solution at comparable or greater concentrations to MAF. After 1 hr, MAF-exposed cells exhibited nominal EC_50_ of 1.59 µg/mL for EA.hy926 cells, while EC_50_ for HepG2 cells was 7.26 µg/mL. Results of cytotoxicity assays exposed for 24 h to the same conditions can be found in Figure S6. PAH parent compounds such as benzo[a]pyrene have been shown to exert cytotoxicity upon metabolic activation to form diol epoxide compounds that are reactive with purine sections of DNA [42]. While cytotoxicity of individual PAH has been reported even in metabolically non-competent cancer cell lines, including HepG2 [60,61] and EA.hy926 [62], effective concentrations were greater than those that can be delivered using dosing methods tested herein. Thus, further experiments are needed to test exposure to cell lines with greater metabolic activity, such as primary human hepatocytes, and to evaluate additional endpoints. Collectively, these studies showed that solvent-free methods are feasible for delivery of diverse PAH using in vitro systems. However, for in vitro-to-in vivo extrapolation studies where analytical precision is needed for quantitation of the concentrations of the components of the mixture in cell culture media, aqueous MAF is a more reliable option due to greater potency, flexibility in exposed amounts, and consistent compound recovery in the media.

## 4. Conclusions

Our data show that MAF is the most efficient (in terms of sample preparation) and consistent (with respect to quantifying constituent concentrations) dosing method for delivering complex substances and mixtures in multi-well plate-based in vitro assays, an acute need in experimental toxicology and regulatory science [17]. While dosing via solvent extraction enabled characterization of compound bioavailability in cell culture media at relatively low exposure levels, exposure from MAF yielded both consistent analytical recovery and greater potency in cytotoxic effects in vitro. This is important to consider upon future application to more complex substances; a higher analytical response greatly facilitates targeted and untargeted chemical characterization of the mixture to inform in vitro-to-in vivo extrapolations [63]. Prior studies found that DMSO extraction selectively extracts aromatic compounds compared to aliphatic compounds and may restrict the bioactive fraction of the complex test substance [16]; additional studies are thus needed to investigate the effectiveness of MAF and micro-O-rings for dosing non-aromatic compounds such as aliphatic, naphthenic, or heteroatomic species at this small scale. Moreover, delivery via micro-O-rings overall yields less quantifiable analytical recovery, an obstacle for quantitative characterization of more complex substances. Since accommodated fraction and O-rings are regularly utilized for aquatic toxicity testing with success, further study is needed to apply and support these dosing methods with realistic complex substances for in vitro assays.

## Data Availability

All pertinent data are included in the Supplementary Materials.

## Supplementary Materials

The following are available online at https://www.mdpi.com/article/10.3390/toxics11010019/s1, Figure S1: Schematic of micro-O-ring kinetics experimental protocol, Figure S2: Micro-O-ring absorption kinetics detailed by aromatic class, Figure S3: Solubility calculations modeled from Fischer et al. 2019 [46], Figure S4: No-cell recovery replicated for 24 h exposure, Figure S5: Recovery of individual PAH tested with and without cells, Figure S6: Cytotoxicity analyses for 24 h exposure, Table S1: Table of chemicals included in this study, Table S2: Retention times for individual PAH and ISTD used for GC-MS/MS method, Table S3: Transitions for GC-MS/MS method, Table S4: Kinetics for micro-O-ring absorption vs. Smith et al. 2010 [24], Table S5: Kinetics for micro-O-ring recovery over time vs. Smith et al. 2010 [24], Table S6: Raw GC-MS/MS data for recovery of compounds with corresponding ISTD.

## Abbreviations

PAH, polycyclic aromatic hydrocarbons; GC-MS/MS, gas chromatography tandem mass spectrometry; MAF, media accommodated fraction; ASTM, American Society for Testing and Materials; DMSO, dimethyl sulfoxide; ECHA, European Chemicals Agency; WAF, water accommodated fraction; OECD, Organization for Economic Cooperation and Development; DMEM, Dulbecco’s Modified Eagle Medium; TAB, tetraoctylammonium bromide; ISTD, internal standard; K_OW_, octanol-water partition coefficient; LOQ, limit of quantification; S/N, signal-to-noise ratio; F_MD_, media dissolved fraction; F_UB_, fraction unbound; TriAr, triaromatic; PolyAr, polyaromatic; DiAr, diaromatic; NDiAr, naphthenic diaromatic.
