# Supplementary material for: Dosing Methods to Enable Cell-Based In Vitro Testing of Complex Substances: A Case Study with a PAH Mixture
Source: Toxics. 2022 Dec 26;11(1):19. doi: 10.3390/toxics11010019 (PMC9866728; doi:10.3390/toxics11010019)
Supplement: Supplementary file 1 [file toxics-11-00019-s001.zip › Supplemental Information Table of Contents.pdf]

Supplemental Information: Table of Contents

Methods:

- Table S1: Table of chemicals included in this study
- Table S2: Retention times for individual PAH and ISTD used for GC-MS/MS method
- Table S3: Transitions for GC-MS/MS method

Figure 2:

- Figure S1: Schematic of micro-O-ring kinetics experimental protocol
- Figure S2: Micro-O-ring absorption kinetics detailed by aromatic class
- Table S4: Kinetics for micro-O-ring absorption vs. Smith et al. 2010

Figure 3:

- Figure S3: Solubility calculations modeled from Fischer et al. 2019
- Table S5: Kinetics for micro-O-ring recovery over time vs. Smith et al. 2010

Figure 4:

- Figure S4: Figure 4 No-cell recovery replicated for 24 hr exposure
- Figure S5: Recovery of individual PAH tested with and without cells
- Table S6: Raw GC-MS/MS data for recovery of compounds with corresponding ISTD

Figure 5:

- Figure S6: Figure 5 Cytotoxicity analyses for 24 hr exposure
