# Supplementary figures and images for: Dosing Methods to Enable Cell-Based In Vitro Testing of Complex Substances: A Case Study with a PAH Mixture
Source: Toxics. 2022 Dec 26;11(1):19. doi: 10.3390/toxics11010019 (PMC9866728; doi:10.3390/toxics11010019)

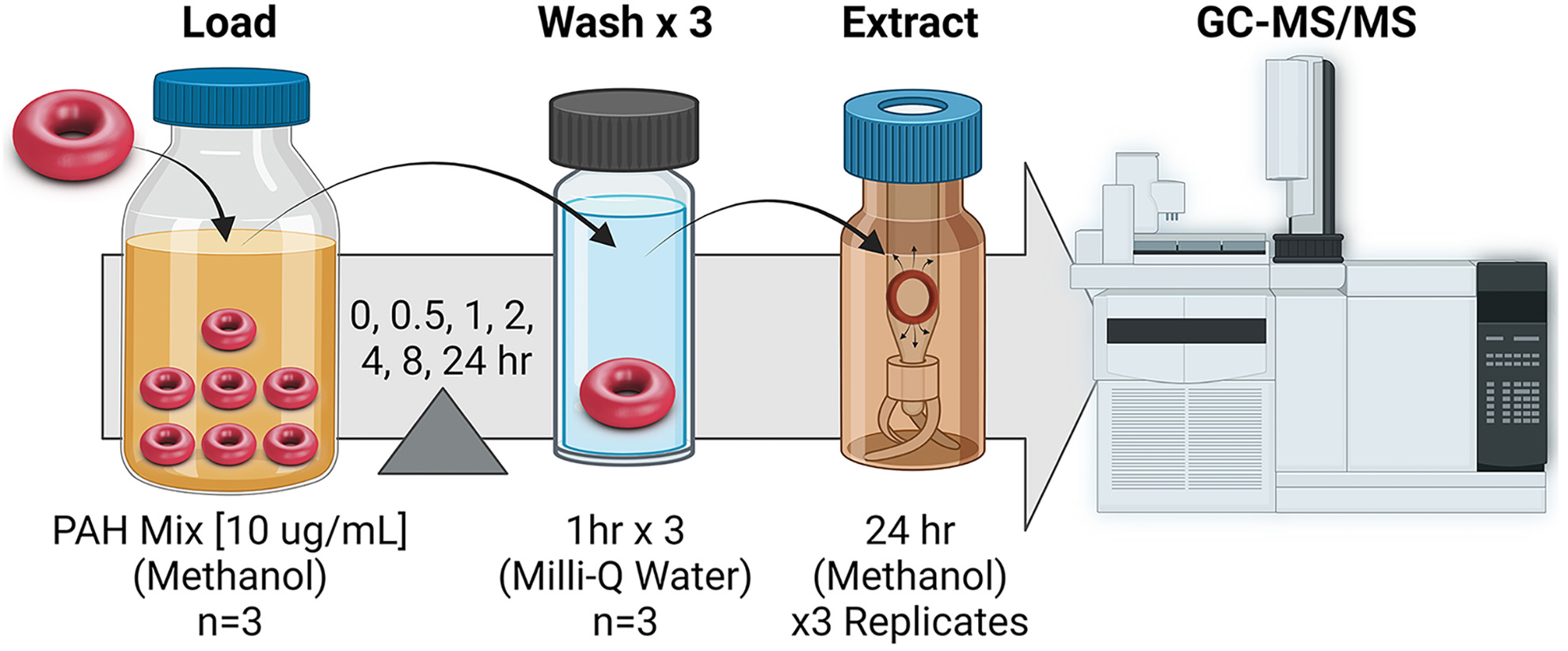

Supplement: Supplementary file 1 [file toxics-11-00019-s001.zip › Figure S1.jpg]

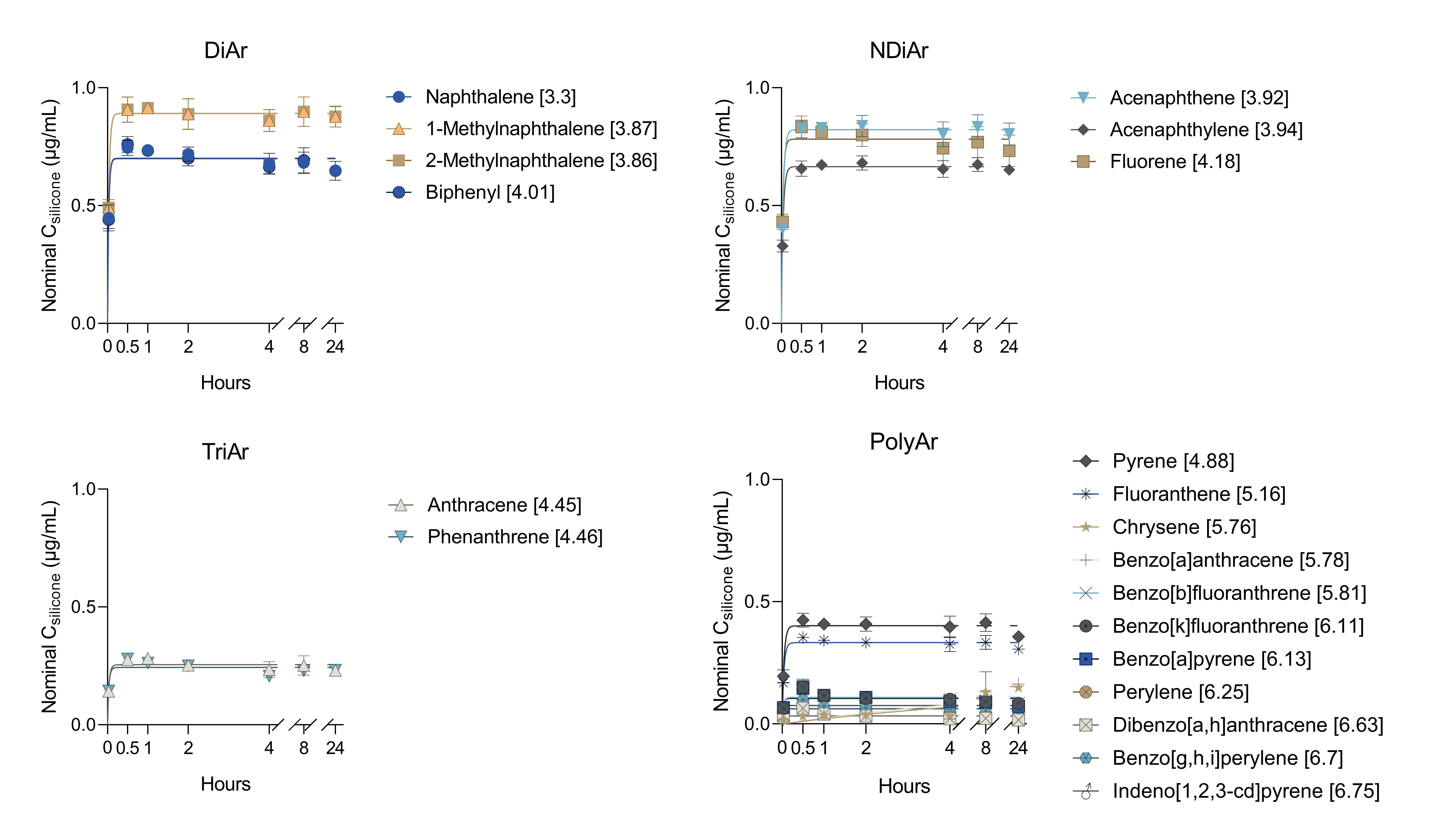

Supplement: Supplementary file 1 [file toxics-11-00019-s001.zip › Figure S2.jpg]

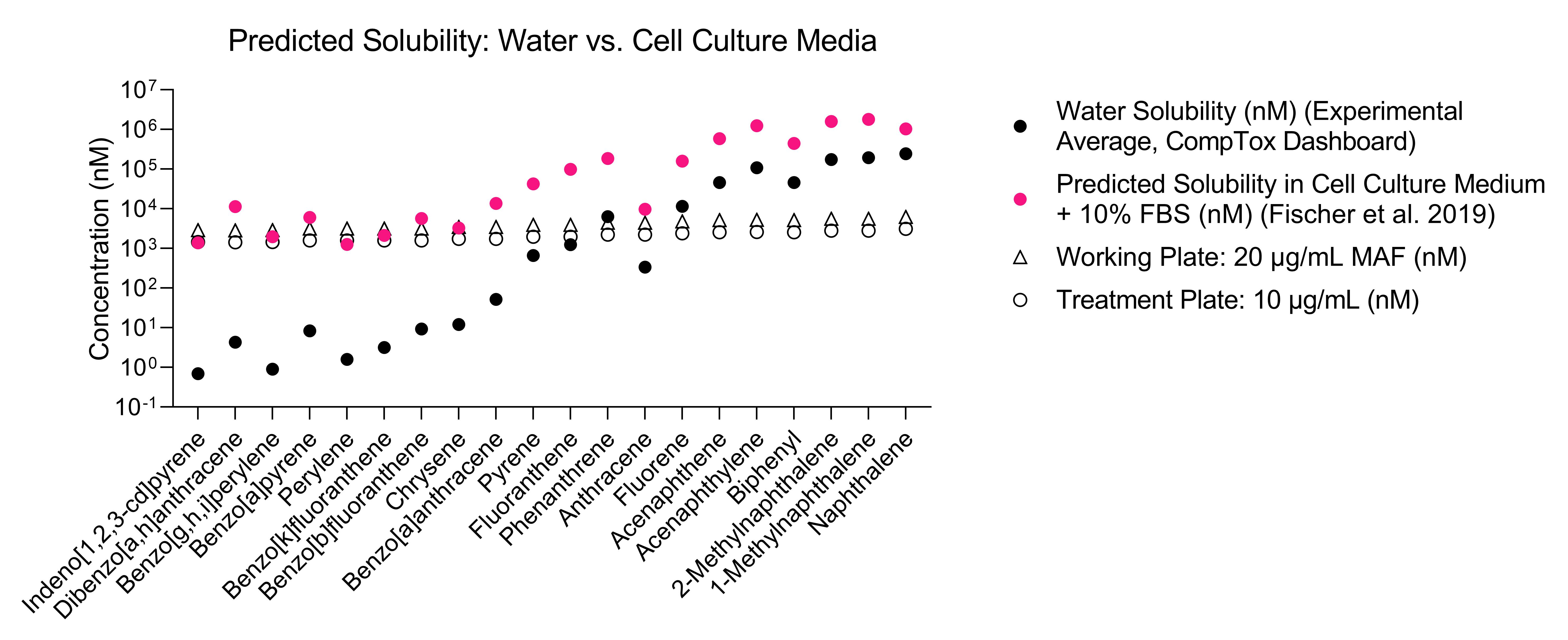

Supplement: Supplementary file 1 [file toxics-11-00019-s001.zip › Figure S3.jpg]

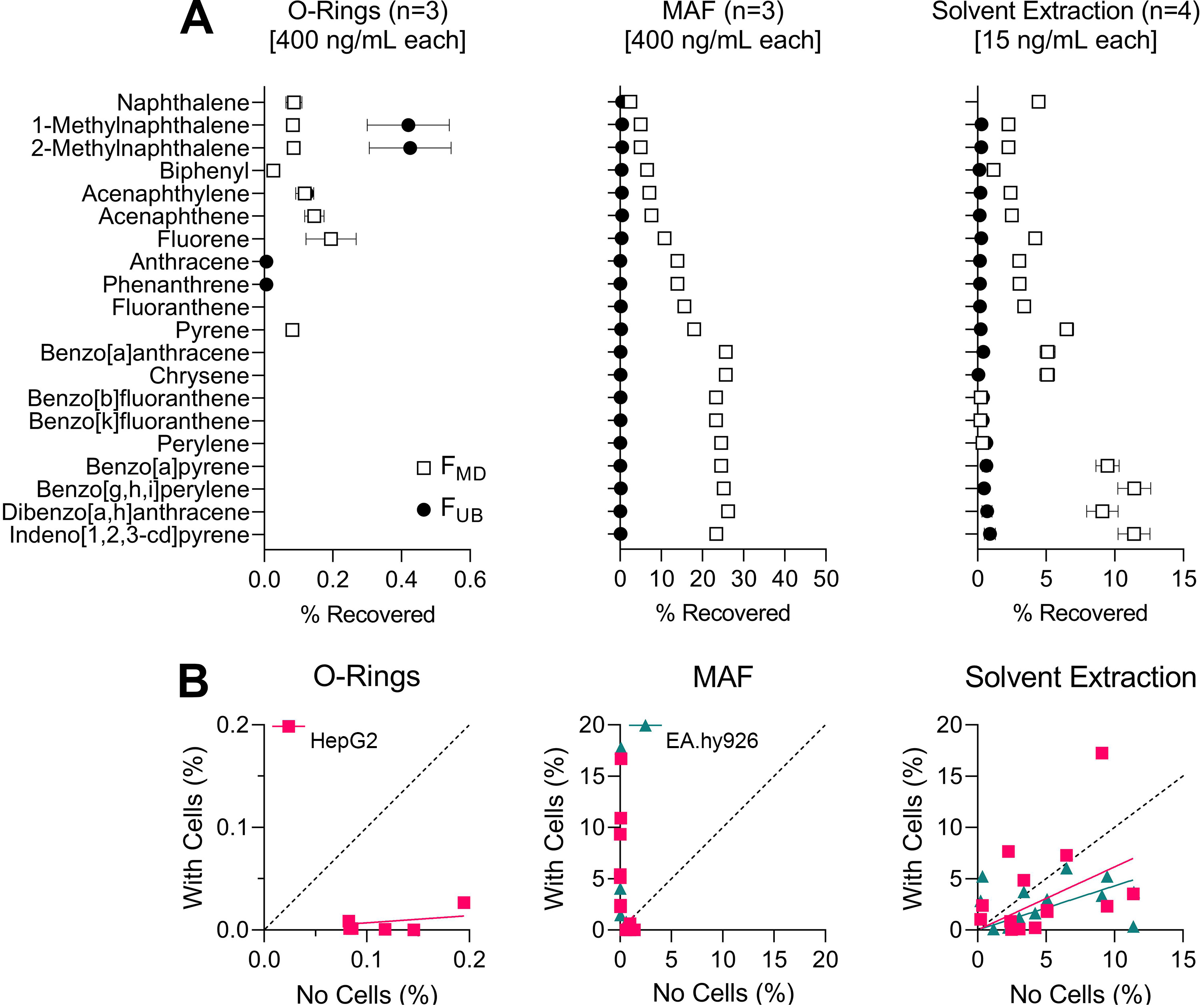

Supplement: Supplementary file 1 [file toxics-11-00019-s001.zip › Figure S4.jpg]

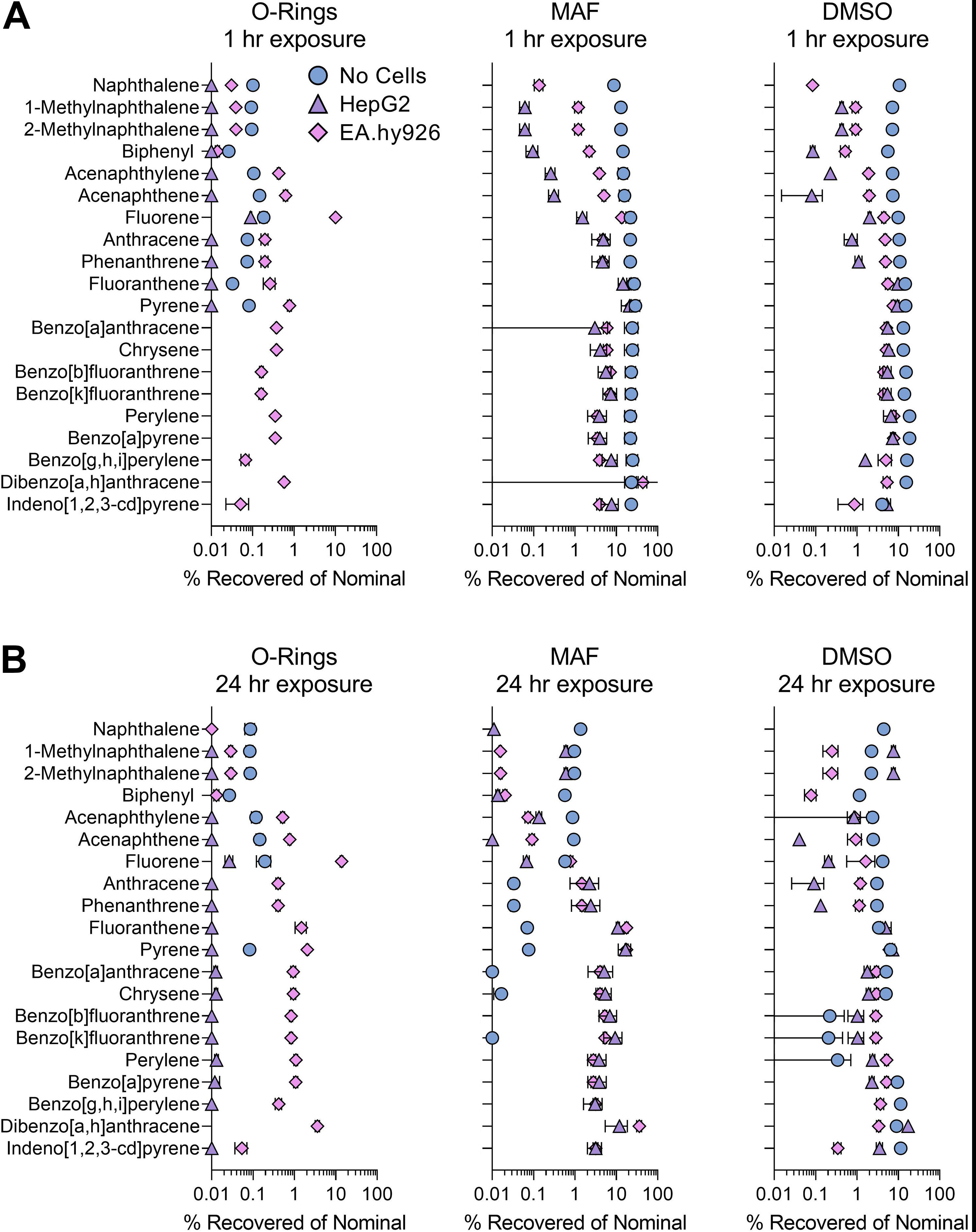

Supplement: Supplementary file 1 [file toxics-11-00019-s001.zip › Figure S5.jpg]

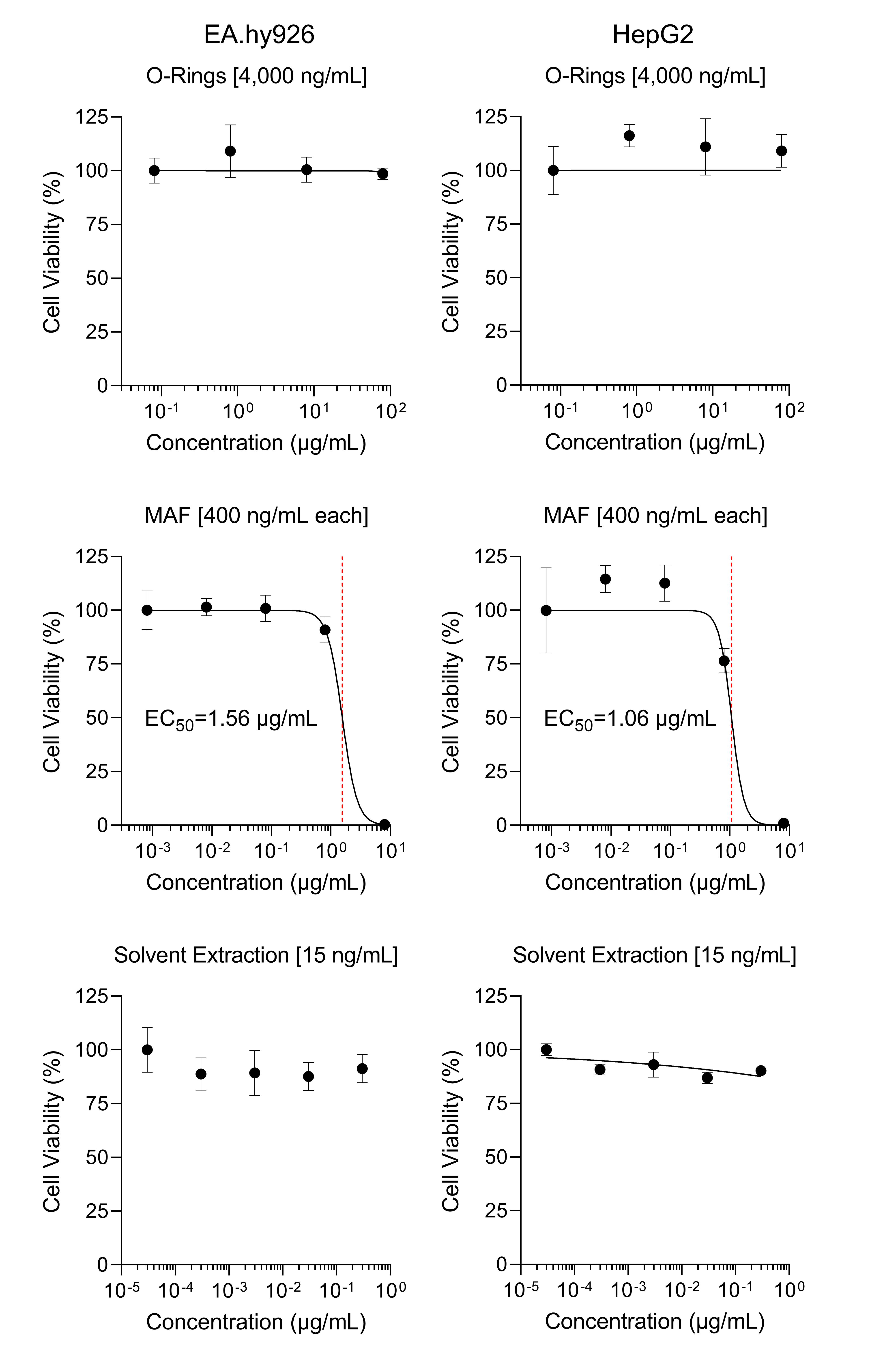

Supplement: Supplementary file 1 [file toxics-11-00019-s001.zip › Figure S6.jpg]
